# Neoadjuvant therapy followed by local excision and two-stage total mesorectal excision: a new strategy for sphincter preservation in locally advanced ultra-low rectal cancer

**DOI:** 10.1093/gastro/got040

**Published:** 2014-01-21

**Authors:** Ting Wang, Jianping Wang, Yanhong Deng, Xiaojian Wu, Lei Wang

**Affiliations:** ^1^Department of Colorectal Surgery, The Sixth Affiliated Hospital (The Gastrointestinal & Anal Hospital) of Sun Yat-sen University, Guangzhou, China and ^2^Department of Oncology, The Sixth Affiliated Hospital of Sun Yat-sen University, Guangzhou, China

**Keywords:** rectal cancer; sphincter-preservation; neoadjuvant therapy; local excision; total mesorectal excision

## Abstract

**Background:** With the increased usage of neoadjuvant chemoradiotherapy, improved surgical technique and stapling devices, sphincter-preserving resection has become more frequent for patients with rectal cancer. However, as for locally advanced ultra-low rectal cancer, sphincter-preservation is still facing an enormous challenge.

**Objective:** To introduce an NLT strategy of sphincter-preservation—neoadjuvant therapy (NT) followed by local excision (LE) and two-stage total mesorectal excision (TME)—into the treatment of locally advanced ultra-low rectal cancer (lesions with anal sphincter invasion).

**Methods:** From October 2010 to October 2011, nine patients with locally advanced rectal cancer located less than 3 cm from the anal verge were treated by the NLT strategy. All patients had shown good clinical response to NT. The LE procedure was carried transanally 6–8 weeks after completion of the NT. TME was performed to dissect mesorectal lymph nodes 4–6 weeks after LE.

**Results:** Of the nine patients, the lesion was assessed as T2 in two, T3 in five, and T4 in two before NT, and lymph node metastasis was detected in five patients. The median distance from the tumor to the anal verge was 2.5 cm (range: 1–3 cm). The median follow-up was 27 months (range: 24–34 months). No distant metastasis was detected. Only one patient (11.1%) developed local recurrence at 12 months post-operatively and then underwent abdomino-perineal resection. The remaining eight patients had preserved long-term continence and the median Wexner score at two years post-operation was 4 (range: 2–6).

**Conclusion:** The new NLT strategy can achieve sphincter-preservation in some patients with ultra-low rectal cancer, with favorable oncological outcome and preservation of normal anal sphincter function.

## INTRODUCTION

Sphincter preservation is one of the main objectives of the surgical treatment of rectal cancer. With the increased usage of neoadjuvant chemoradiotherapy (nCRT), improved surgical technique and stapling devices, sphincter-preserving resection has become more frequent in patients with rectal cancer. Long-term follow-up results of a Dutch trial showed that nCRT induced tumor regression and perirectal lymph node sterilization, leading to a significantly improved local control of Stage II and -III rectal cancer, compared with total mesorectal excision (TME) alone [1]. Neoadjuvant CRT, followed by TME and sphincter-preserving procedures after 6–8 weeks, is the standard care for Stage II and Stage III rectal cancer [2].

Since a distal margin of 2 cm was considered adequate for the resection of the rectal wall in the 1980s [3, 4], the 2 cm distal rule between the tumor and the anal ring has been widely accepted, which has resulted in all rectal cancers lying more than 2 cm from the anal ring or more than 5 cm from the anal verge being treated by sphincter-preserving procedures. The technique of inter-sphincteric resection (ISR) further permits us to achieve conservative surgery in patients with a tumor close to- or in the anal canal without compromising local control and long-term survival [5]. However, it may be associated with worse functional outcomes and morbidity risks following internal sphincter excision and colo-anal anastomosis [6–7].

With the improvement of living standards and health awareness, sphincter-preservation in Chinese patients is increasingly desirable. Also, the age of onset of rectal cancer in the Chinese population is younger than that in the western populations, and low rectal cancer accounts for a high proportion of rectal cancer cases in China [8], which presents a greater challenge in terms of sphincter-preservation for Chinese colorectal surgeons. As for ultra-low rectal cancer—that is, lesions with internal anal sphincter invasion—we herein proposed an NLT strategy of sphincter-preservation: neoadjuvant therapy (NT) followed by local excision (LE) and two-stage TME. The aim of the present study was to evaluate the feasibility and safety of NLT strategy for ultra-low rectal cancer after NT.

## PATIENTS AND METHODS

### Patient Selection

Patients were collected from a multicenter prospective randomized controlled trial (FOWARC trial, NCT01211210), started in October 2010, which compared neoadjuvant FOLFOX6 chemotherapy with or without radiation in locally advanced rectal cancer.

The inclusion criteria for the present study were patients with ultra-low rectal cancers (locally advanced ultra-low rectal cancers located less than 3 cm from the anal verge or less than 1 cm from the dentate line) who showed good response to NT but still could not be treated by sphincter-preserving resection through multi-disciplinary team (MDT) evaluation. As in the selection of the operative procedure during multi-disciplinary team evaluation, the distance of the lesion from the anus, the patients' comorbidities, and the condition of the anal sphincter were considered. Patients who were excluded from the study were (i) those with T1, T2N0 and M0 who did not undergo NT, (ii) those responded badly to NT and were treated by an abdomino-perineal resection (APR), (iii) those who showed good response to NT and were treated by sphincter-preserving procedures, e.g. anterior resection or ISR and (iv) those who were followed up for less than two years.

Tumor location was defined as the lowest distal part of the tumor. Clinical tumor staging was performed before NT according to the 7^th^ edition of the AJCC Staging Manual [9]. Endorectal ultrasound was used to determine the invasive depth of the tumor and pelvic magnetic resonance imaging was used to determine the anal sphincter involvement and regional lymph node metastasis. Clinical response to NT was assessed by endorectal ultrasound and pelvic magnetic resonance imaging six week after completion of the neoadjuvant therapy. Good response was defined as tumor volume reduction of more than 50% or T stage downgrade. Pathological tumor staging of the resected specimen was also performed in accordance with the guidelines of the AJCC Staging Manual [9]. Circumferential resection margin (CRM) was scored as positive when cancer cells were within 1 mm of the margin. Evidence of complete pathological response (pCR) was defined as absence of viable adenocarcinoma in the surgical specimen or the presence of lakes of mucus without tumor cells.

### NLT treatment strategy

Neoadjuvant CRT was used as recommended by both the NCCN and ESMO guidelines [10]. This consisted of 45 grays (Gy) administered in fractions of 1.8 Gy over a five-week period using three fields, megavoltage (18 megavolts). The treated volume included the mesorectum and internal iliac nodes up to S1 and S2, and the entire anal canal with the anal verge was included inferiorly. Patients also received 5 cycles of pre-operative concomitant FOLFOX6 chemotherapy, which was oxaliplatin 85 mg/m^2^, leucovorin 400 mg/m^2^ and bolus plus infusional 5-FU 2.8 mg/m^2^.

Informed consent was obtained from all patients before surgery, with an explanation of the potential risks of increased disease recurrence and reduced survival associated with LE. The LE procedure was carried out via a transanal approach or by transanal endoscopic microsurgery under spinal or general anesthesia 6–8 weeks after completion of the NT. The patients were placed in a prone jackknife or lithotomy position, depending on the tumor location. The residual tumor was removed by full-thickness excision of rectal wall with a 1 cm margin around the tumor. The rectal defect was closed primarily in a transverse fashion with absorbable sutures.

**Figure 1.**
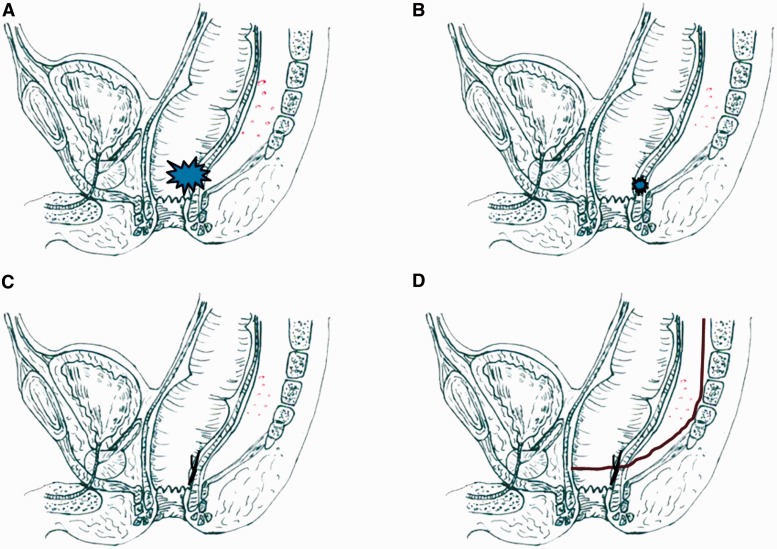
NLT strategy for locally advanced ultra-low rectal cancer. (A) Primary tumor with internal sphincter involvement. (B) Tumor regression and down-stage after NT. (C) Local excision 6–8 weeks after NT. (D) Two-stage total mesorectal excision 4–6 weeks after local excision.

TME was performed 4–6 weeks after LE when the anal incision had completely healed. The rectum was mobilized in an anterior and posterior manner with sharp dissection through Denonvilliers' and Waldeyer's fascia. Lateral dissection of the rectum was employed, in order not to breach the fascia propria of the rectum, remaining outside the margins of the mesorectum. The rectum was transected at the level of the anorectal ring with a transverse stapler. A circular stapler of a suitable size was applied to perform the double stapled colorectal anastomosis. The complete NLT strategy is shown in Figure 1. The Ethics and Research Committee of Sun Yat-sen University approved the study protocol and the data collection.

### Follow-up

Patients were scheduled for follow-up visits every three months during the first two years and every six months thereafter. Follow-up evaluations included physical examination, digital rectal examination, and measurement of the serum carcino-embryonic antigen level. Abdominal and pelvic computed tomography and chest radiography were performed every six months for the first two years and yearly thereafter. Colonoscopy was performed annually during the first three years. Abnormal physical findings or laboratory results mandated further screening as indicated, based on the clinicians’ decisions. Local recurrence was defined as any recurrence that was diagnosed or suspected in the pelvis (anastomosis, tumor bed, or pelvic nodes) occurring alone or with distant metastases six months after resection. The Wexner fecal incontinence score was used to evaluate anal function and a score >7 was considered as fecal incontinence [11]. The last follow-up date was 30 September 2013. Time to last follow-up or death was measured from the date of TME procedure.

## RESULTS

**Figure 2.**
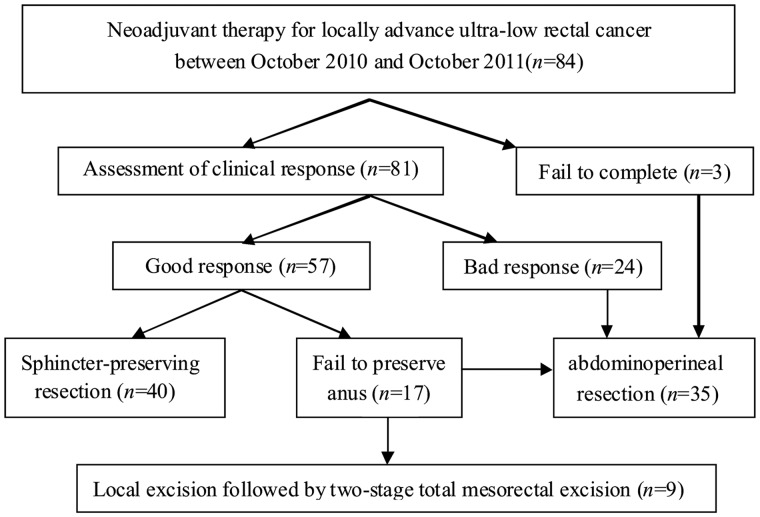
Selection procedure for patients treated by NLT strategy.

A total of 84 patients with locally advanced ultra-low rectal cancer underwent NT from October 2010 to October 2011. Eighty-one of them finished the NT as planned, among whom 51 (63.0%) showed good clinical response. Through MDT evaluation, 17 patients were not considered for sphincter-preserving procedures, among whom nine had a strong desire for sphincter-preservation despite MDT decision dictated otherwise, and accepted the NLT treatment strategy. The selection procedure for these patients is summarized in Figure 2.

Clinical and pathological characteristics, treatment and outcomes of the nine patients are reported in Table 1. The median distance from the tumor to the anal verge was 2.5 cm (range: 1–3 cm). Clinical staging before NT was T2N+ in two patients, T3N0 in three, T3N+ in two, T4N0 in one, and T4N+ in one. All the patients showed definite T stage downgrade with tumor regression after NT, among whom three (33.3%) showed clinical complete response (cCR). Five patients underwent transanal excision and four had the excision performed by transanal endoscopic microsurgery. Negative CRM was acquired in all patients. Urinary retention occurred in one patient (11.1%) after LE, and was treated by Foley catheter placement for three days. The median interval between LE and TME was 4.5 weeks (range: 4–6 weeks). The median number of lymph nodes harvested during TME was 17 (range: 13–23). There was no major post-operative complication following TME. The final pathological report showed T0N0 (pathological complete response, pCR) in two patients (22.2%), T0N1 in two, T1N1 in two, T2N0 in one, T2N1 in two.

**Table 1.** Tumor characteristics, treatment and outcomes of the nine patients treated according to the NLT strategy

Case	Age (years) /sex	Tumor size (cm)	Distance from anal verge (cm)	Tumor grade	Clinical stage	NT protocol	Tumor size for post-NT (cm)	Clinical stage for post-NT	LE procedure	CRM	Number of lymph nodes dissected during TME	Pathological stage for post-op	Post-op Compli- cations	Follow-up (months)	Post-op relapse	Wexner Score at 2 years post-op
1	62/male	2.5	2.5	Ⅱ	T3N0	CT	0.5	T1N0	Transanal	(-)	15	T1N0	None	34; survive	None	3
2	44/male	5.0	1.0	Ⅲ	T2N+	CRT	2.0	T1N+	Transanal	(-)	23	T1N1	None	31; survive	None	4
3	65/male	3.5	3.0	Ⅲ	T4N0	CT	1.5	T2N0	TEM	(-)	17	T2N0	Urinary retention	30; survive	None	4
4	63/female	3.0	2.5	Ⅰ	T3N0	CRT	0	T0N0	Transanal	(-)	15	T0N0	None	28; survive	None	6
5	54/male	3.5	1.5	Ⅱ	T4N+	CT	1.5	T2N0	TEM	(-)	17	T2N1	None	27; survive	None	3
6	48/female	2.0	2.5	Ⅱ	T3N+	CRT	0	T0N+	Transanal	(-)	19	T1N1	None	27; survive	None	5
7	35/male	4.0	2.0	Ⅱ	T3N+	CRT	2.0	T2N+	TEM	(-)	21	T2N1	None	26; survive	Local relapse	-
8	59/female	2.5	1.5	Ⅲ	T2N+	CRT	0	T0N0	Transanal	(-)	14	T0N0	None	25; survive	None	5
9	39/male	4.5	2.5	Ⅰ	T3N0	CT	0.5	T1N0	TEM	(-)	18	T1N0	None	24; survive	None	2

NT = neoadjuvant therapy; CT = chemotherapy; CRT = chemoradiotherapy; LE = local excision; TEM = transanal endoscopic microsurgery; CRM = circumferential resection margin; TME = total mesorectal excision; post-op = post-operative

The median follow-up period was 27 months (range: 24–34 months). No distant metastasis was detected and one patient (11.1%) had local recurrence 12 months after surgery. The patient underwent APR and was alive at the date of the final follow-up. The remaining eight patients retained long-term continence and the median Wexner score at two years post-operation was 4 (range: 2–6).

## DISCUSSION

Traditionally, all rectal tumors positioned at less than 5 cm from the anal verge or less than 2 cm from the anal ring have been treated by ARP. In the Dutch trial including 1805 patients with rectal cancer, the rate of sphincter preservation in rectal cancer was 66%, suggesting that most low rectal cancers were treated by APR [1]. Despite the emergence of ISR results in a greater proportion of sphincter-preserving procedures, concern over impairment of anal function limits its widespread use. The modern treatment of low rectal cancer calls for successful eradication of the disease and preservation of a normal anal sphincter function with a good quality of life. The objective of our study was to demonstrate that, by complying with an NLT strategy, most patients with ultra-low rectal cancer were able successfully to undergo a sphincter-preserving procedure without compromising the oncological outcome or anal sphincter function. The nine patients who were considered as requiring APR were treated by LE and two-stage TME following NT, with good local tumor control (only one relapsed) and satisfactory anal function (no fecal incontinence occurred).

The benefits of nCRT have been well documented, including tumor regression and down-staging associated with increased tumor resectability and a higher rate of sphincter preservation. After nCRT, pCR is observed in 8–27% of patients and has been associated with reduced rates of local recurrence and distant metastases and improved survival at 5 years after TME surgery [12]. Another recent trend in rectal cancer management is a renewed interest in LE, which has been recommended in the Chinese Standard for the Diagnosis and Treatment of Colorectal Cancer (2010) for the treatment of early stage (T1N0) rectal cancer with favorable histology because of the low risk of regional spread [13]. With the routine use of NT, LE was tested for locally advanced rectal cancer in several centers, with as good oncological results as TME [14–28].

Although LE following NT has recently become a more attractive option for locally advanced rectal cancer, it remains controversial, mainly because mesorectal lymph nodes may be involved. By further analysing the previous studies, we found that the excellent oncological results were acheived largely due to those with pCR to neoadjuvant therapy. In most of those studies, the local recurrence rate of patients with pCR was zero [14–18, 21, 22, 25, 26, 28]. Therefore, LE alone following NT can be used only for patients with pCR [29, 30]. However, the identification of patients with true pCR to NT is very challenging, due to lack of definitive tumor staging and difficulty in predicting the status of mesorectal lymph nodes.

The aim of our study was to preserve the anus and anal function without compromising local control and survival in patients with locally advanced ultra-low rectal cancer, rather than pursuing minimally invasive surgery and decreasing morbidity. In our study, most patients did not have pCR to NT or mixed lymph node metastasis, so two-stage TME was performed 4–6 weeks after LE to dissect mesorectal lymph nodes. Since pre-operative irradiation induced thickening of the pre-sacral fascia, the TME procedure could be performed safely with no significant increase in bleeding.

In the present study, all the nine patients treated by NLT strategy preserved the anus and survived at least two years. Only one patient (pT2N1) relapsed and only one patient developed urinary retention. CRM has been shown to be a more important oncological factor than distal margin in rectal cancer surgery [5]. All the patients with ultra-low rectal cancer acquire negative CRM. This may be due to the use of long-course NT, which reduces the tumor volume and transforms vegetative lesions into ulcerative scar that facilitates surgery and decreases tumor spillage. These results indicate that an NLT treatment strategy can achieve excellent oncological safety whilst simultaneously preserving the anus.

Impairment of the anal sphincter with nCRT has been a matter of concern. The effects of irradiation on ano-rectal function are dose-dependent [31]. In the present study, patients who underwent neoadjuvant chemotherapy seemed to have lower Wexner score (median 3, range 2–4) than those with nCRT (median 5, range 4–6). Hopefully the statistically significant difference in anal function between neoadjuvant chemotherapy and nCRT could be testified in the FOWARC trial (NCT01211210) conducted by our institute. In addition, for low rectal cancer, sphincter-preserving surgery itself has some impact on anal function. After being treated in accordance with the NLT strategy, our patients preserved long-term continence and obtained a good quality of life, which benefited from preservation of the internal sphincter and avoidance of colo-anal anastomosis during two-stage TME, following the removal of the tumor by LE.

Several studies have reported that patients with cCR undergoing a non-operative (wait and see) strategy appear to achieve similar oncological results, compared with patients with pCR after TME [32–34]. However, assessment of tumor response to CRT is challenging. Hiotis *et al.* performed a retrospective analysis of 488 locally advanced rectal cancer patients, treated by CRT followed by TME, and found that 75% of cCR patients had residual cancer in the surgical specimen (residual T2 or T3 cancer in 60% of patients), with nodal metastases in 18% of cases [35]. Guillem *et al.* confirmed in a prospective study that digital rectal examination and proctoscopy are inaccurate methods of determining tumor response to CRT, with underestimated evaluation in 78% of cases and a correct identification of a pCR in only 21% of patients [36]. In our study, following NT, two patients (22.2%) were T-under-staged and one N-under-staged. These unsatisfactory clinical and radiological assessment results for tumor response to CRT may be explained by the distribution of residual cancer cells in the layers of the rectal wall after CRT [37]. Hence TME remains the standard of care for rectal cancer, even in patients with cCR, while a wait-and-see strategy or an LE alone should be offered only in the context of a clinical trial, or reserved for highly selected patients (e.g. well/moderately differentiated adenocarcinoma with no evident perirectal lymph node metastases).

The new NLT strategy makes it possible for most ultra-low rectal cancers to benefit from sphincter-preserving resection and the choice between a sphincter-preserving procedure and APR is not related to the distance from the tumor to the anal verge or the anal ring. For rectal cancer at ≤3 cm from the anal verge, the achievement of long-term survival and low local recurrence rate, without compromising anal function, supports this new NLT treatment strategy; however, it must be prudently introduced into current management of ultra-low rectal cancer. Large prospective and randomized clinical trials with long follow-up are needed to assess the oncological efficacy of NLT strategy, compared with APR.

## FUNDING

This study was supported by the Programme of Introducing Talents of Discipline to Universities (No. B12003), and National Natural Science Foundation of China (No. 81101669)

**Conflict of interest:** none declared.

## References

[1] van Gijn W, Marijnen CA, Nagtegaal ID, Dutch Colorectal Cancer Group (2011). Pre-operative radiotherapy combined with total mesorectal excision for resectable rectal cancer: 12-year follow-up of the multicentre, randomised controlled TME trial. Lancet Oncol.

[2] Valentini V, Aristei C, Glimelius B, Scientific Committee (2009). Multidisciplinary rectal cancer management: 2nd European rectal cancer consensus conference (EURECAcc2). Radiother Oncol.

[3] Williams NS, Dixon MF, Johnston D (1983). Reappraisal of the 5 centimetre rule of distal excision for carcinoma of the rectum: a study of distal intramural spread and of patients’ survival. Br J Surg.

[4] Pollett WG, Nicholls RJ (1983). The relationship between the extent of distal clearance and survival and local recurrence rates after curative anterior resection for carcinoma of the rectum. Ann Surg.

[5] Rullier E, Laurent C, Bretagnol F (2005). Sphincter-saving resection for all rectal carcinomas: the end of the 2 cm distal rule. Ann Surg.

[6] Tilney HS, Tekkis PP (2008). Extending the horizons of restorative rectal surgery: intersphincteric resection for low rectal cancer. Colorectal Dis.

[7] Martin ST, Heneghan HM, Winter DC (2012). Systematic review of outcomes after intersphincteric resection for low rectal cancer. Br J Surg.

[8] Xu AG, Yu ZJ, Jiang B (2010). Colorectal cancer in Guangdong Province of China: a demographic and anatomic survey. World J Gastroenterol.

[9] American Joint Committee on Cancer: AJCC Cancer Staging Handbook.

[10] Sauer R, Becker H, Hohenberger W (2004). Pre-operative versus post-operative chemoradiotherapy for rectal cancer. N Engl J Med.

[11] Jorge JM, Wexner SD (1993). Etiology and management of fecal incontinence. Dis Colon Rectum.

[12] Maas M, Nelemans PJ, Valentini V (2010). Long-term outcome in patients with a pathological complete response after chemoradiation for rectal cancer: a pooled analysis of individual patient data. Lancet Oncol.

[13] Ministry of Health of the People’s Republic of China (2010). Chinese Standard for the Diagnosis and Treatment of Colorectal Cancer. Chin J Gastroint Surg.

[14] Schell SR, Zlotecki RA, Mendenhall WM (2002). Transanal excision of locally advanced rectal cancers down-staged using neoadjuvant chemoradiotherapy. J Am Coll Surg.

[15] Ruo L, Guillem JG, Minsky BD (2002). Pre-operative radiation with or without chemotherapy and full-thickness transanal excision for selected T2 and T3 distal rectal cancers. Int J Colorectal Dis.

[16] Bonnen M, Crane C, Vauthey JN (2004). Long-term results using local excision after pre-operative chemoradiation among selected T3 rectal cancer patients. Int J Radiat Oncol Biol Phys.

[17] Stipa F, Zernecke A, Moore HG (2004). Residual mesorectal lymph node involvement following neoadjuvant combined-modality therapy: rationale for radical resection?. Ann Surg Oncol.

[18] Caricato M, Borzomati D, Ausania F (2006). Complementary use of local excision and transanal endoscopic microsurgery for rectal cancer after neoadjuvant chemoradiation. Surg Endosc.

[19] Borschitz T, Wachtlin D, Mohler M (2008). Neoadjuvant chemoradiation and local excision for T2–3 rectal cancer. Ann Surg Oncol.

[20] Nair RM, Siegel EM, Chen DT (2008). Long-term results of transanal excision after neoadjuvant chemoradiation for T2 and T3 adenocarcinomas of the rectum. J Gastrointest Surg.

[21] Huh JW, Jung EJ, Park YA (2008). Pre-operative chemoradiation followed by transanal excision for rectal cancer. J Surg Res.

[22] Kundel Y, Brenner R, Purim O (2010). Is local excision after complete pathological response to neoadjuvant chemoradiation for rectal cancer an acceptable treatment option?. Dis Colon Rectum.

[23] Callender GG, Das P, Rodriguez-Bigas MA (2010). Local excision after pre-operative chemoradiation results in an equivalent outcome to total mesorectal excision in selected patients with T3 rectal cancer. Ann Surg Oncol.

[24] Belluco C, De Paoli A, Canzonieri V (2011). Long-term outcome of patients with complete pathological response after neoadjuvant chemoradiation for cT3 rectal cancer: implications for local excision surgical strategies. Ann Surg Oncol.

[25] Lezoche G, Guerrieri M, Baldarelli M (2011). Transanal endoscopic microsurgery for 135 patients with small non-advanced low rectal cancer (iT1–iT2, iN0): short- and long-term results. Surg Endosc.

[26] Issa N, Murninkas A, Powsner E (2012). Long-term outcome of local excision after complete pathological response to neoadjuvant chemoradiation therapy for rectal cancer. World J Surg.

[27] Tennyson N, Mendenhall WM, Morris CG (2012). Transanal excision with radiation therapy for rectal adenocarcinoma. Clin Med Res.

[28] Yu CS, Yun HR, Shin EJ (2013). Local excision after neoadjuvant chemoradiation therapy in advanced rectal cancer: a national multicenter analysis. Am J Surg.

[29] Dademade G, Wexner SD (2012). Complete response after neoadjuvant therapy in rectal cancer: to operate or not to operate?. Dig Dis.

[30] Fichera A, Allaix ME (2013). Paradigm-shifting new evidence for treatment of rectal cancer. J Gastrointest Surg.

[31] Canda AE, Terzi C, Gorken IB (2010). Effects of pre-operative chemoradiotherapy on anal sphincter functions and quality of life in rectal cancer patients. Int J Colorectal Dis.

[32] Habr-Gama A, Perez RO, Nadalin W (2004). Operative versus non-operative treatment for stage 0 distal rectal cancer following chemoradiation therapy. Long-term results. Ann Surg.

[33] Maas M, Beets-Tan RG, Lambregts DM (2011). Wait-and-see policy for clinical complete responders after chemoradiation for rectal cancer. J Clin Oncol.

[34] Smith JD, Ruby JA, Goodman KA (2012). Nonoperative management of rectal cancer with complete clinical response after neoadjuvant therapy. Ann Surg.

[35] Hiotis SP, Weber SM, Cohen AM (2002). Assessing the predictive value of clinical complete response to neoadjuvant therapy for rectal cancer: an analysis of 488 patients. J Am Coll Surg.

[36] Guillem JG, Chessin DB, Shia J (2005). Clinical examination following pre-operative chemoradiation for rectal cancer is not a reliable surrogate end point. J Clin Oncol.

[37] Duldulao MP, Lee W, Streja L (2013). Distribution of residual cancer cells in the bowel wall after neoadjuvant chemoradiation in patients with rectal cancer. Dis Colon Rectum.

